# Retention force of polyetheretherketone and cobalt-chrome-molybdenum removable dental prosthesis clasps after artificial aging

**DOI:** 10.1007/s00784-020-03642-5

**Published:** 2020-10-16

**Authors:** Felicitas Mayinger, Danka Micovic, Andreas Schleich, Malgorzata Roos, Marlis Eichberger, Bogna Stawarczyk

**Affiliations:** 1 Department of Prosthetic Dentistry, University Hospital, LMU Munich, Goethestraße 70, 80336 Munich, Germany; 2 Department of Biostatistics, Epidemiology, Biostatistics and Prevention Institute, University of Zurich, Hirschengraben 84, 8001 Zurich, Switzerland

**Keywords:** PEEK, Cobalt-chrome-molybdenum, Clasp, Removable dental prosthesis, Retention force

## Abstract

**Objectives:**

To examine the retention force of removable dental prosthesis (RDP) clasps made from polyetheretherketone (PEEK) and cobalt-chrome-molybdenum (CoCrMo, control group) after storage in water and artificial aging.

**Materials and methods:**

For each material, 15 Bonwill clasps with retentive buccal and reciprocal lingual arms situated between the second pre- and first molar were manufactured by milling (Dentokeep [PEEKmilled1], NT digital implant technology; breCAM BioHPP Blank [PEEKmilled2], bredent), pressing (BioHPP Granulat for 2 press [PEEKpressed], bredent), or casting (remanium GM 800+ [CoCrMo], Dentaurum); *N* = 60, *n* = 15/subgroup. A total of 50 retention force measurements were performed for each specimen per aging level (initial; after storage [30 days, 37 °C] and 10,000 thermal cycles; after storage [60 days, 37 °C] and 20,000 thermal cycles) in a pull-off test. Data were statistically analyzed using one-way ANOVA, post hoc Scheffé and mixed models (*p* < 0.05).

**Results:**

Initial, PEEKpressed (80.2 ± 35.2) and PEEKmilled1 (98.9 ± 40.3) presented the lowest results, while PEEKmilled2 (170.2 ± 51.8) showed the highest values. After artificial aging, the highest retention force was observed for the control group (131.4 ± 56.8). The influence of artificial aging was significantly higher for PEEK-based materials. While PEEKmilled2 and PEEKpressed showed an initial decline in retention force, all other groups presented no impact or an increase in retention force over a repetitive insertion and removal of the clasps.

**Conclusions:**

Within the tested PEEK materials, PEEKmilled2 presented superior results than PEEKpressed. Although CoCrMo showed higher values after artificial aging, all materials exhibited sufficient retention to recommend usage under clinical conditions.

**Clinical relevance:**

As RDPs are still employed for a wide range of indications, esthetic alternatives to conventional CoCrMo clasps are sought.

## Introduction

Removable dental prostheses (RDPs) are commonly used to treat patients with large or multiple edentulous areas. Indications furthermore include the replacement of missing teeth in patients with severely damaged periodontal tissue, an excessive loss of alveolar bone limiting the possibility for implantation or the use as interim restorations for patients awaiting extensive treatments like bone augmentation [1–3]. In addition, psychological and financial factors play an important role in choosing between RDPs and alternative treatment options like multi-unit fixed dental prostheses (FDPs) or implants.

RDPs are usually manufactured of a PMMA base with acrylic or ceramic teeth in combination with cobalt-chrome-molybdenum (CoCrMo) clasps. The tried and tested CoCrMo clasps show excellent mechanical properties, such as a promising long-term stability and reliability [4–8] and high retentive capabilities, even when manufactured in small dimensions to improve patient comfort [4]. However, CoCrMo’s silver color is nowadays becoming more and more unacceptable for patients with high esthetic requirements, especially when employed in the visible region. Moreover, the biocompatibility of metal clasps is viewed as controversial [9–11]. In the oral cavity, non-precious metals like CoCrMo can cause galvanic corrosions as metallic ions solved in saliva interact with amalgam or gold restorations [12]. In this context, patients have specified a metallic taste in connection with a new removable prosthesis manufactured of CoCrMo or shown allergic reactions of the oral mucosa [9–11],

These disadvantages called for the implementation of new dental compositions such as high-performance thermoplastic polymers as clasp materials in the treatment with RDPs. Polyetheretherketone (PEEK), a member of the polyaryletherketone (PAEK) family, possesses a high biocompatibility, excellent mechanical characteristics, a high chemical stability, and a high temperature resistance [13–16]. Due to its high flexibility, PEEK RDPs induce less stress on abutment teeth and may be less prone to deformation or fracture than standard alloy counterparts [17, 18]. PEEK furthermore possesses a low weight, an important factor for RDPs of the maxilla, and allows for an individual adaption of the clasp color to the patients’ natural tooth color. As of today, PEEK materials are available in a multitude of shades, from classic pearl white to a wide variety of different enamel colors. To reduce extensive surgical procedures for FDP treatment of patients presenting with deficiencies of soft and hard tissues in the esthetic zone and enable RDPs to be manufactured solely from PEEK, a pale-pink shade option has been developed to imitate the color of the gum. A recent case report describing the long-term outcome of a treatment with a PEEK RDP has observed the patient to perceive this restoration as more acceptable and easier to assimilate to than alloy alternatives [19]. PEEK materials are nowadays employed for a wide range of restorations in prosthetic dentistry, from dental implants, abutments, FDPs, frameworks of RDPs to clasps, or telescopic prostheses [20–22]. In implant dentistry, flexible PEEK frameworks can reduce excessive masticatory forces occurring due to a lack of proprioception [23]. PEEK restorations can be produced employing the conventional lost-wax technique by pressing from pellets or granules, or via computer-aided design and computer-aided manufacturing (CAD/CAM) by milling from blanks. The use of CAD/CAM allows for a fully digital workflow that entails numerous advantages like an increased material homogeneity and the ability to reproduce restorations, for example, when elderly patients misplace their prostheses.

One property of utmost importance for a clasp is its retention force, which will keep the dental prosthesis in place during function such as eating or speaking. This point strongly affects the patients’ contentment with their restoration. One way to measure retention force in an in vitro study set-up is the pull-off test, where specimens are removed from abrasion-resistant models under constant measurement conditions.

The aim of the present study was thus to examine the retention force of clasps made from different PEEK materials in comparison with a CoCrMo control group after storage in water and artificial aging with thermocycling. The study tested the null hypothesis that neither the clasp material, the manufacturing process of the PEEK specimens, artificial aging nor a repetitive insertion and removal of the clasps on an abrasion-resistant CoCrMo model showed an impact on the retention force.

## Materials and methods

**Fig. 1:**
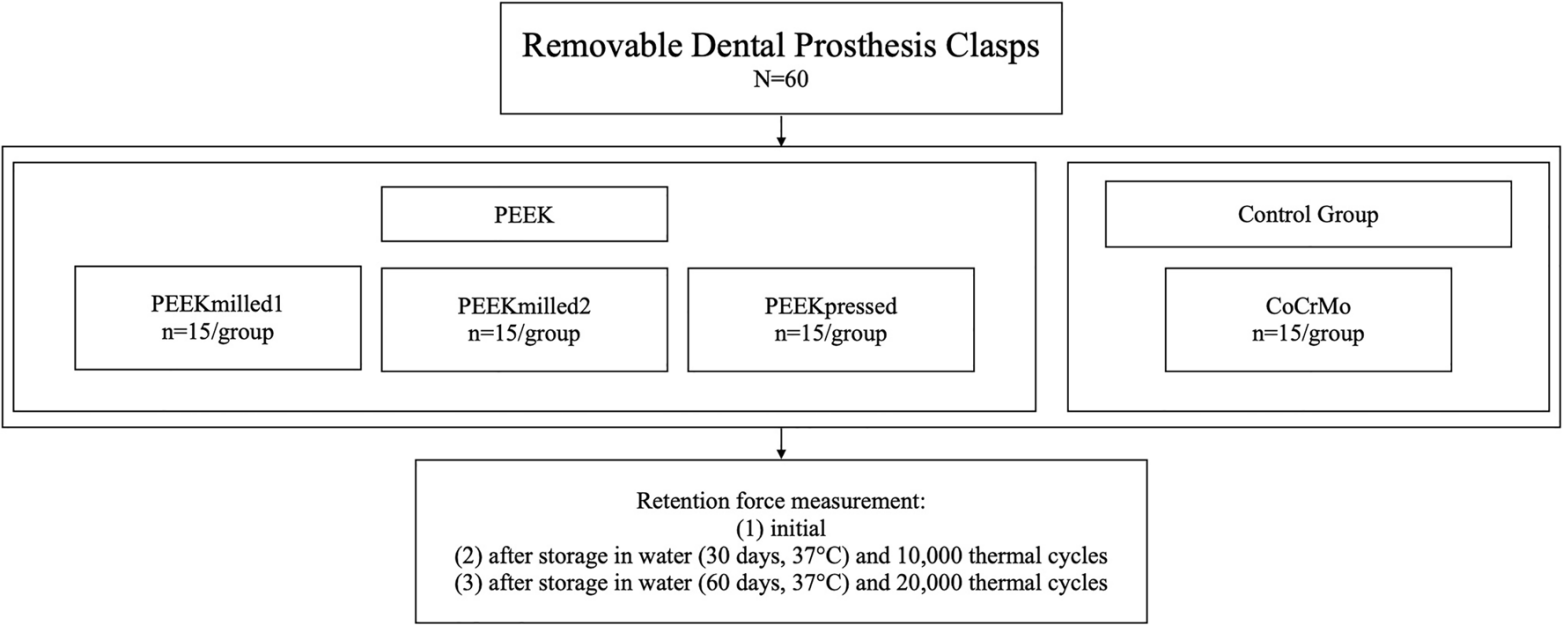
Study design

The retention force of clasps made from three differently manufactured PEEK materials (Dentokeep [abbreviation: PEEKmilled1], NT digital implant technology, Karlsruhe, Germany; breCAM BioHPP Blank [PEEKmilled2] and BioHPP Granulat for 2 press [PEEKpressed], bredent, Senden, Germany) and a CoCrMo alloy (control group; remanium GM 800+ [CoCrMo], Dentaurum, Ispringen, Germany) was examined in a pull-off test at different aging levels (Table 1 and Fig. 1).

**Table 1:** Materials, abbreviations, manufacturers, compositions, and lot. no. used

Material	Abbreviations	Shade	Manufacturers	Compositions	Lot. no.
Dentokeep	PEEKmilled1	Pearl white	NT digital implant technology, Karlsruhe, Germany	Polyether ether ketone, inorganic fillers (20%)	11DK18001
breCAM BioHPP Blank	PEEKmilled2		bredent, Senden, Germany		380149
BioHPP Granulat for 2 press	PEEKpressed				379806
Remanium GM 800+	CoCrMo		Dentaurum, Ispringen, Germany	Co (58.3%), Cr (32.0%), Mo (6.5%), W (1.5%), Si (1.0%)	816

### Specimen fabrication

**Fig. 2:**
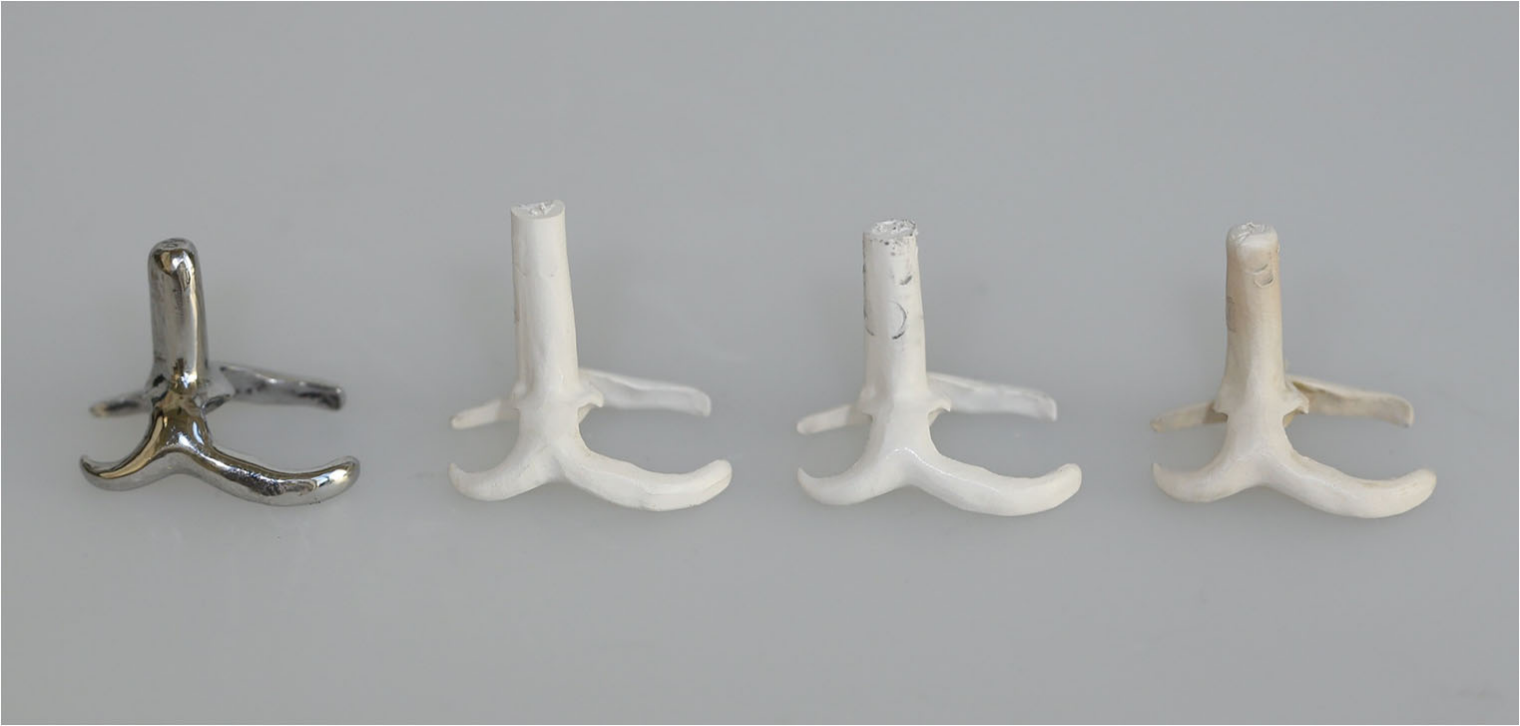
RDP clasp specimens made of CoCrMo, PEEKmilled1, PEEKmilled2 and PEEKpressed

For each material, 15 specimens were manufactured (*N* = 60; *n* = 15/subgroup; Fig. 2).

The second pre- and first molar of a dental arch model (Frasaco Mandible 119, A-3, Franz Sachs & Co, Tettnang, Germany) were prepared to incorporate a Bonwill clasp. A master clasp was produced from CoCrMo (remanium GM 800+) by casting (Globucast, Krupp AG, Essen, Germany) with the lost-wax technique (Finowax, DT, Bad Kissingen, Germany). The casting channel, which was positioned in the insertion direction of the Bonwill clasp, was cut to a height of 15 mm to allow for a later positioning in the pull-off test. The specimen was air-particle abraded (basis Quattro IS, Renfert, Hilzingen, Germany) with 110 μm Al_2_O_3_ (Korox 110, Bego, Bremen, Germany) at 0.2 MPa and subsequently polished with a silicone polisher and a polishing brush (Komet, Gebr. Brasseler GmbH & Co. KG, Lemgo, Germany) before scanning (Ceramill map V2.5.02, Amann Girrbach, Koblach, Austria) was performed to create a master STL file (Table 2).

**Table 2:** Dimensions of the Bonwill clasp

	Length (mm)	Height (mm)	Width (mm)	Undercut (mm)
Retentive arm, overall (external dimension)	19.0			
Reciprocal arm, overall (external dimension)	16.2			
Retentive arm, short (inner dimension)	4.9	2.33	1.76	0.75
Retentive arm, long (inner dimension)	10.5	2.9	1.72	1.0
Reciprocal arm, short (inner dimension)	5.5	1.79	1.73	
Reciprocal arm, long (inner dimension)	8.7	2.91	1.89	
Support		2.0	4.8	
Connector			4.5 × 4.92	

Retentive arm (buccal), reciprocal arm (lingual), short arm (premolar), and long arm (molar)

Clasps made of PMMA (Zeno® PMMA cast Disc, Wieland Dental + Technik, Pforzheim, Germany; *n* = 30) and PEEK (Dentokeep and breCAM BioHPP Blank; n = 15/subgroup) were then manufactured with CAM software (Zenotec CAM, V2.2.009, Wieland Dental + Technik) using a milling machine (i-Mes 4030, Wieland Dental + Technik).

**Fig. 3:**
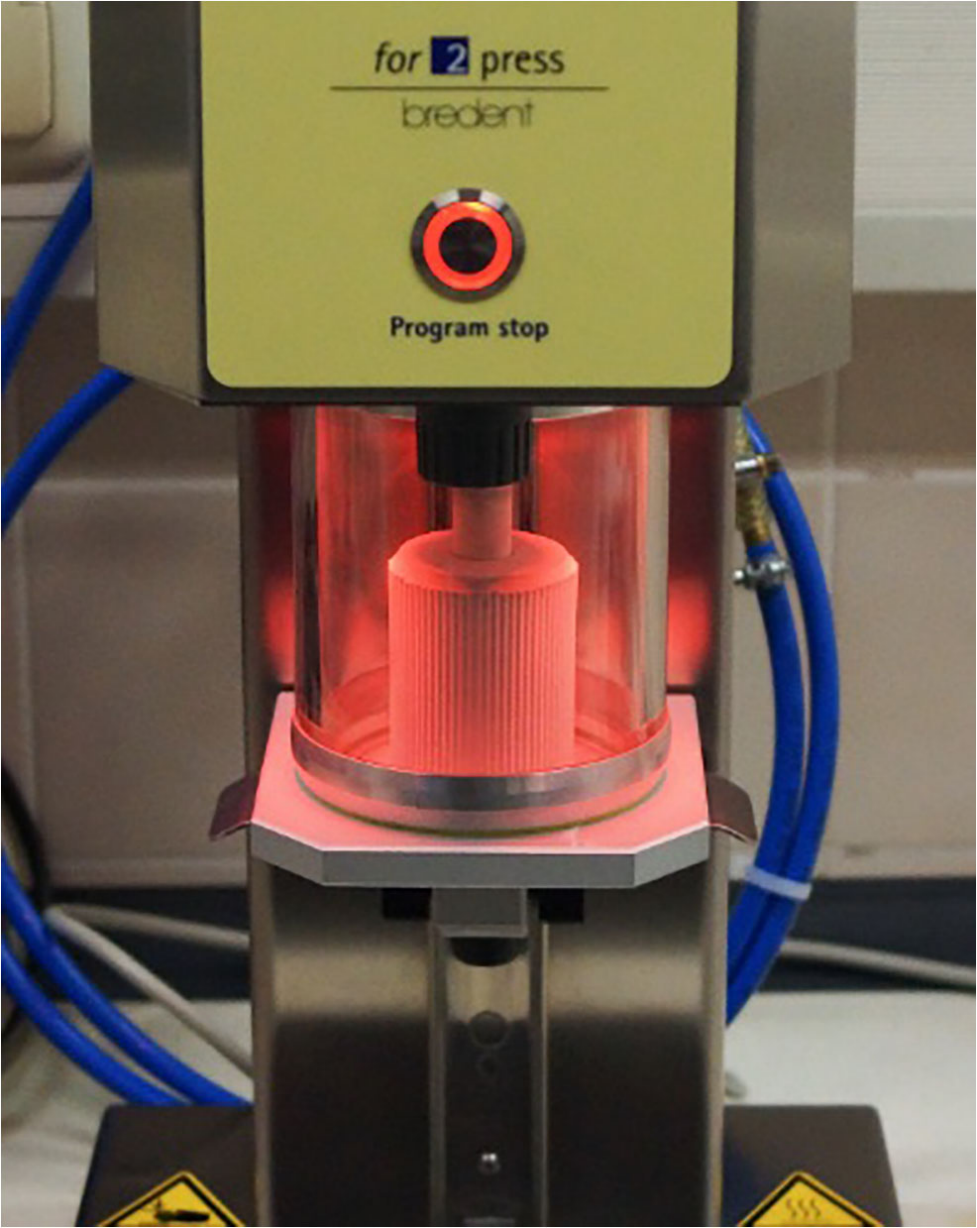
Pressing process for clasps made from PEEKpressed (for 2 press, bredent)

PEEKpressed specimens were produced by carefully embedding the PMMA clasps (Brevest for 2 press, bredent). The investment ring was then heated closely following the manufacturer’s instructions (ARCA 20, Schütz Dental, Rosbach, Germany) and Granulat was pressed under vacuum (for 2 press, bredent; Fig. 3).

Following the same workflow, CoCrMo specimens (remanium GM 800+) were produced by embedding PMMA clasps (JET2000, Siladent, Dr. Böhme & Schöps GmbH, Goslar, Germany). The investment ring was then heated closely following the manufacturer’s instructions (KaVo EWL 5636, KaVo Dental GmbH, Biberach/Riß, Germany) before clasps were cast at 1410 °C with a pressure of 0.45 MPa (Globucast).

After outbedding, PEEKpressed and CoCrMo specimens were air-particle abraded with 105 μm Al_2_O_3_ at 0.2 MPa (Hasenfratz, Fine-blaster type FG 3, Sandmaster, Zofingen, Switzerland).

Connectors and casting channels were cut to a height of 15 mm before specimens were polished with a goat hairbrush and buffing wheel using polishing paste (Universal-Polierpaste, Ivoclar Vivadent, Ellwangen, Germany). All specimens were then fitted on CoCrMo models using occlusion foil (Hanel Okklusions-Folie 12 μm, Coltène/Whaledent AG, Altstätten, Switzerland).

### Measurement of the retention force

Retention force was determined at different aging levels:

1. Initial,
2. After storage in distilled water for 30 days at 37 °C in an incubator (Hera Cell 150, Heraeus, Hanau, Germany) and artificial aging with 10,000 thermal cycles (Thermocycler THE-1100, SD Mechatronik, Feldkirchen-Westerham, Germany), with specimens remaining in each bath set to 5 °C and 55 °C for 20 s, simulating 1 year in clinical conditions [24], and
3. After storage in distilled water for 60 days at 37 °C and artificial aging with 20,000 thermal cycles (Thermocycler THE-1100) simulating a clinical period of 2 years.

**Fig. 4:**
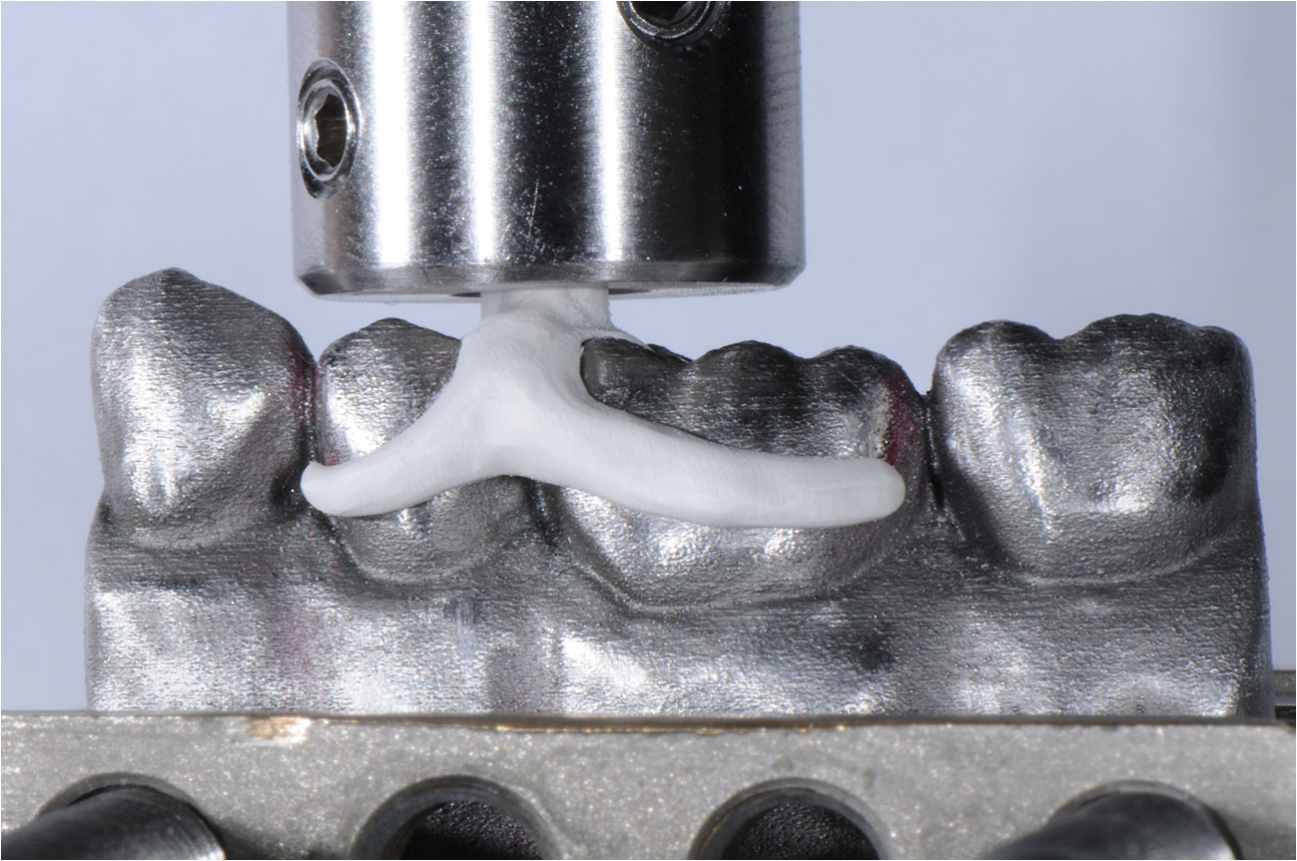
Retention force measurement (Zwick 1445, Zwick GmbH & Co. KG)

For the pull-off test, models were carefully positioned in the insertion direction before casting channels/connectors were inserted in an individually manufactured stainless steel adapter (SD Mechatronik GmbH, Feldkirchen, Germany; Fig. 4). Pull-off force was applied with a crosshead speed of 5 mm per minute employing the universal testing machine (Zwick 1445, Zwick GmbH & Co. KG, Ulm, Germany) until the maximum force dropped by 10%. For each specimen, 50 measurements were performed at the three different aging levels.

### Statistical analysis

Prior to performing this study, a power analysis had been computed using nQuery Advisior (Version 6.04.10, Statistical Solutions, Saugaus Mass, USA). For this calculation, retention force values of the control group (163 ± 55 N) were used. A sample size of 15 in each group would have a power of 97% to detect a difference of 81.5 N using a two-group *t* test with a significance level of *α* = 0.05. The Bonferroni correction would furthermore have a power of 92% under identical conditions.

Statistical evaluation of the data was performed with descriptive analysis followed by Kolmogorov-Smirnov for testing the violation of normal distribution. One-way ANOVA followed by the Scheffé post hoc test was performed to determine the influence of the material and aging level on the retention force. To determine global retention force values within the tested groups and potential changes of these values at different aging levels and measurement intervals, as each clasp was measured 50 times leading to dependent measurements, linear mixed models were computed.

All *p* values below 0.05 were construed as statistically significant. Data were analyzed with SPSS version 25.0 (IBM, Armonk, NY, USA).

## Results

The results of the descriptive analyses are presented in Table 3. As no violation of normality assumption was indicated, parametric tests were performed.

**Table 3:** Descriptive statistics for the retention force [N] of the different clasp materials at varying aging levels

Aging level	PEEKmilled1		PEEKmilled2		PEEKpressed		CoCrMo	
	Mean ± SD	95% CI	Mean ± SD	95% CI	Mean ± SD	95% CI	Mean ± SD	95% CI
1. Initial	98.9 ± 40.3^a,b^	[76.6; 121.3]	170.2 ± 51.8^c^	[141.5; 199.0]	80.2 ± 35.2^a^	[60.6; 99.7]	139.7 ± 57.4^b,c^	[107.8; 171.5]
2. After storage in water (30 days, 37 °C) and 10,000 thermal cycles	76.3 ± 27.9^a^	[60.8; 91.8]	134.2 ± 44.0^b^	[109.7; 158.6]	63.2 ± 26.4^a^	[48.5; 77.9]	147.6 ± 54.8^b^	[117.2; 178.0]
3. After storage in water (60 days, 37 °C) and 20,000 thermal cycles	50.3 ± 21.2^a,b^	[38.5; 62.1]	80.0 ± 31.4^b^	[62.6; 97.4]	41.2 ± 14.0^a^	[33.3; 49.0]	131.4 ± 56.8^c^	[99.9; 162.9]

^a,b,c^Different letters present significant differences between the different materials within one aging level

The clasp material showed an influence on the retention force (*p* < 0.001). Initial, PEEKpressed and PEEKmilled1 showed the lowest values, while PEEKmilled2 presented the highest results. The control group led to results in the same value range as both PEEKmilled1 and PEEKmilled2. After artificial aging with storage in water (30 days, 37 °C) and 10,000 thermal cycles, PEEKpressed and PEEKmilled1 presented significantly lower retention force values than PEEKmilled2 and CoCrMo. After additional artificial aging (storage in water [60 days, 37 °C] and 20,000 thermal cycles), PEEKpressed and PEEKmilled1 showed lower retention force values than the control group, while PEEKmilled2 presented results in the same value range as PEEKmilled1.

Initially, values for PEEKmilled1 (9.5 N [0.0; 18.5]; *p* = 0.04) and CoCrMo (11.2 N [8.9; 13.4]; *p* < 0.001) increased over the repetitive insertion and removal of the clasps on the abrasion-resistant CoCrMo models, while PEEKmilled2 (− 2.9 N [− 4.3; − 1.5]; *p* < 0.001) and PEEKpressed (− 3.1 N [− 4.3; − 2.0]; *p* < 0.001) showed a decline in retention force. After the first artificial aging level, all groups but PEEKpressed that showed a rise in retention force (2.9 N [2.2; 3.6]; *p* < 0.001) showed no impact of a repeated insertion and abrasion on the retention force. After artificial aging with 60-day storage in water at 37 °C and 20,000 thermal cycles, all groups presented an increase in retention force (PEEKmilled1: 6.1 N [5.4; 6.7]; PEEKmilled2: 13.6 N [13.0; 14.3]; PEEKpressed: 5.0 N [4.5; 5.6], CoCrMo: 18.8 N [17.3; 20.4]; *p* < 0.001) over the repetitive insertion and removal of the clasps.

Mixed models defining the control group as baseline showed no significant difference between CoCrMo and PEEKmilled2 (*p* = 0.051) initial, while PEEKmilled1 (− 44.2 N [− 73.8; − 14.6]; *p* = 0.004) and PEEKpressed (− 62.7 N [− 92.2; − 33.1]; *p* < 0.001) presented lower retention force values.

**Fig. 5:**
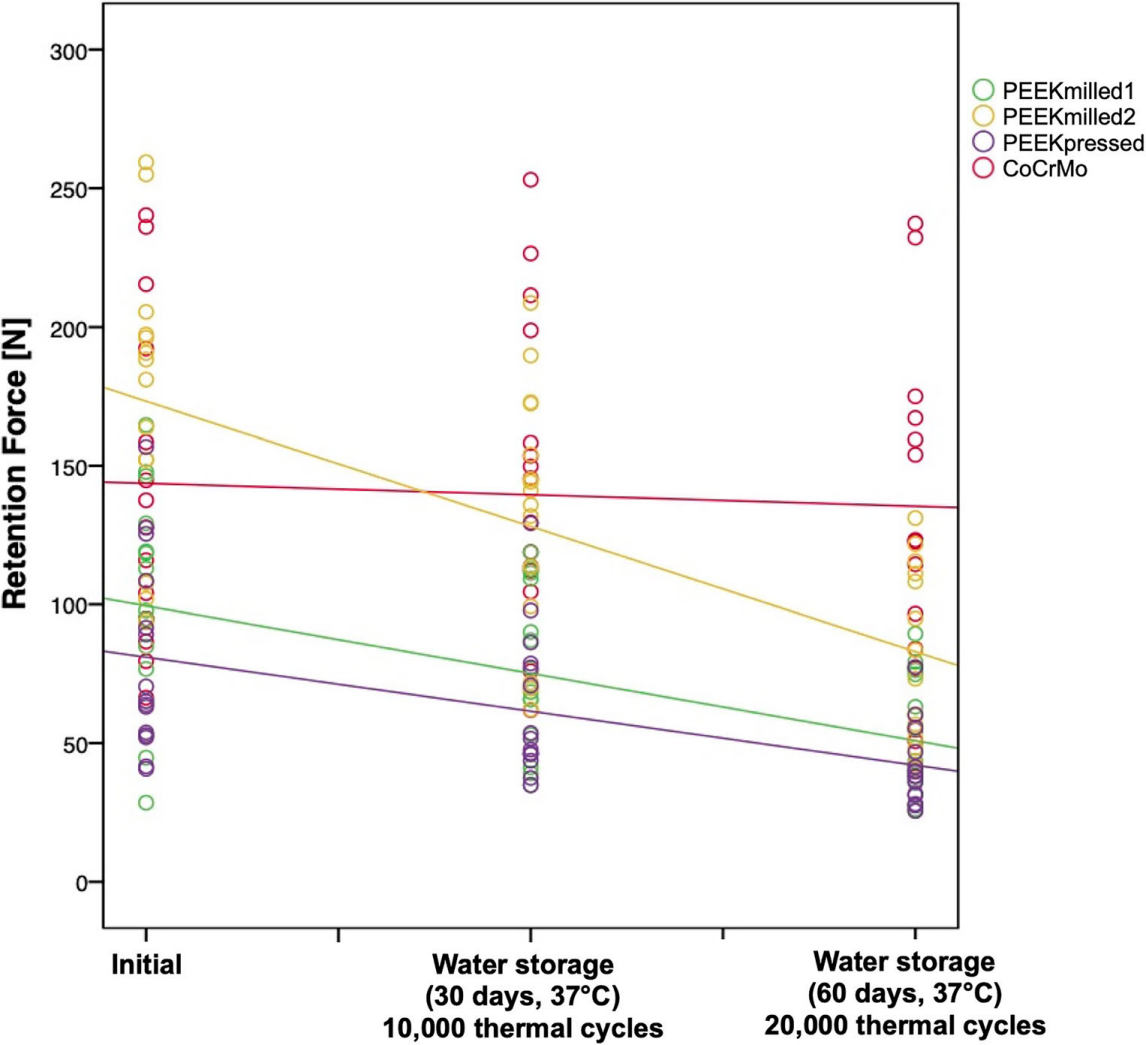
Depiction of the influence of artificial aging on the retention force [N] of all tested materials

The influence of artificial aging was significantly higher for PEEK-based materials (PEEKmilled1: − 20.2 N [− 27.7; − 12.6]; PEEKmilled2: − 41.0 N [− 48.5; − 33.4]; PEEKpressed: − 15.4 N [− 22.9; − 7.8]; *p* < 0.001) than for the control group (Fig. 5).

## Discussion

The aim of this study was to examine the retention force of clasps made from different PEEK materials in comparison with a CoCrMo control group after storage in water and artificial aging with thermocycling to approximate a clinical situation. The tested null hypothesis had to be rejected, as the choice of material, artificial aging, and the repetitive insertion and removal of the clasps on the abrasion-resistant CoCrMo model showed an impact on the retention force.

The present study observed PEEK clasps to present significantly lower retention force values than CoCrMo after artificial aging. In a recent study, the mean retention force of PEEK clasps (2.06–3.67 N) was also reported to be smaller than values observed for CoCr (8.25 N) [17]. As the aspired retention force per clasp has, however, been described as 5–10 N [25, 26], a clinical application of PEEK clasps may be cautiously recommended [27]. Yet, one crucial parameter in this context is stress phenomena occurring during the insertion and removal of RDP clasps. With the choice of material dictating the clasp design, flexible PEEK can require a deeper undercut to ensure sufficient retention force [17]. During removal, high stress levels may thus exceed the strength of the material itself [28]. Further studies are necessary to determine in how far PEEK can represent a clinically valid alternative to established alloy clasps and define an optimum clasp design for this material group.

When regarding the different PEEK materials, it can be reported that PEEKmilled2 presented higher values than PEEKpressed. This might be explained by the differing manufacturing process. While PEEKmilled2 specimens were fabricated from standardized blanks using CAD/CAM technology, clasps pressed from Granulat are more prone to outside influences and application errors, as this manufacturing process entails intricate steps such as the initial embedding, heating, and cooling of the muffle, pressing of the heated material under vacuum, or the subsequent air-abrasion. Following the different steps of this manufacturing process might thus result in an impaired homogeneity of the material [29]. Moreover, the fabrication process of PEEK blanks and PEEK Granulat differs, as PEEK blanks undergo an industrial prepressing procedure, which could increase the mechanical properties of the final product [29]. Contrary to expectations, PEEKmilled1 and PEEKmilled2 presented disparate results in the initial stage. A possible explanation for this puzzling observation may be provided by variations in the industrial manufacturing of the prepressed blanks. After artificial aging, results for the two groups did, however, align. Future investigations are needed to examine this point further.

Although PEEKmilled2 presented higher values than the control group initial, the observed values declined in the course of artificial aging. Artificial aging with 20,000 thermal cycles is supposed to correspond to a clinical situation after 2 years in vivo [24]. The present findings are in agreement with the results of a recently published study that reported specimens milled from PEEK blanks to show decreased mechanical properties after artificial aging [30]. Even though CoCr clasps are reported to show a permanent deformation after aging, they still present higher retention force values than resin clasps due to their high material stiffness and elastic modulus [5].

While PEEKmilled2 and PEEKpressed showed an initial decline in retention force, all other groups presented no impact or an increase in retention force over the repetitive insertion and removal of the clasps on the abrasion-resistant CoCrMo models at the different aging levels. A decline in retention force might be explained by an occurring material fatigue of the PEEKmilled2 and PEEKpressed clasps. Due to PEEK’s low elastic modulus (4 GPa) in comparison with a CoCrMo alloy (240 GPa), it may not be rigid enough to withstand the occurring forces during a repetitive insertion and removal [31]. To counteract this, PEEK clasps could be manufactured to be bulkier and designed with a greater undercut to provide sufficient retentive force [31]. For CoCrMo, the effect of fatigue is seen controversial. While some studies observed a decrease in retention force due to a permanent deformation of the alloy [5], others showed no impact of aging on the retentive values [31]. This might be explained by the differing study set-up, where specimens were rigidly fixed and compromising torqueing forces were thus aimed to be excluded [31]. An increase in retention force, especially for PEEKmilled2 and PEEKpressed specimens that previously showed a decline of the retention force, is, however, unexpected. One possible explanation might be that the repetitive insertion and removal of the clasp specimens entails a better fit through either a minor abrasion of the model or an improved adaption of the clasps through the removal of any imperfections on the inside of the clasp arms. This idea has been described in a previous study, where an increased friction between the two components due to the wear phenomena of the materials was observed in the initial phase of a repetitive insertion and removal of the claps, while an increased wear and decreased retention was reported later on [31].

As of today, only few clinical case reports documenting the behavior of PEEK clasps in vivo are available. One study with a 2-year follow-up showed promising results regarding retention force, color stability, and plaque affinity [19]. The use of PEEK clasps can thus contribute to a healthier periodontium, an important factor for periodontally damaged dentitions, as the low plaque affinity prevents bacterial adhesion [21], while PEEK’s high flexibility entails a low stress on the abutment teeth [17]. These advantages are mirrored in the high satisfaction of both patient and clinician in terms of functional and esthetic results [32]. PEEK clasps can furthermore preserve the existing dentition, with a clinical report describing an absence of scoring phenomena on silicate ceramic or enamel surfaces that are routinely seen for CoCrMo clasps [33]. The low weight of PEEK prostheses, combined with the tooth-similar color and appropriate fit and retention can make these restorations easy to assimilate to [32].

When regarding the findings of the present investigation, PEEK’s promising results during the repetitive insertion and removal of the clasps, and its overall sufficiently high retention force, even after artificial aging, have to be noted. The mechanical properties of PEEK RDP clasps might thus allow the many advantages to be gained from its manufacturing process, from a fully digitalized workflow to a standardized manufacturing process entailing a high material homogeneity. As future material compositions might lead to improved mechanical properties, especially in regard to PEEK’s poor performance in the course of artificial aging, this technique could behold a promising future. The present findings do, however, have to be seen in regard to their limitations, as this in vitro study only examined a limited number of tested materials. Moreover, the rigid model used in this study does not represent the clinical situation accurately, where the periodontal ligament permits a minor flexibility of the natural tooth. As the retention force correlates with the friction coefficient, the different friction coefficients of human enamel, dental restorative materials such as silicate ceramics, and the metallic model employed in the present study have to be considered [34]. This underlines the importance of an individual planning of the clasp geometry, as both the abutment and clasp material hold a decisive impact on the necessary undercut [17, 34]. The microscopical analysis of wear features could provide additional information on the observed differences between PEEK groups [35]. Thus, clinical studies with a long-term follow-up investigating a wider range of PEEK materials are warranted.

## Conclusions

Within the limitations of this study, the following conclusions can be drawn:

1. Within the tested PEEK materials, PEEKmilled2 presented superior results than PEEKpressed.
2. Artificial aging led to a significant decline in retention force for all PEEK-based materials.
3. Overall, groups presented an increase in retention force due to a repetitive insertion and removal of the clasps.
4. Although CoCrMo showed higher values after artificial aging, all materials exhibited sufficient retention to recommend usage under clinical conditions.
